# Factorial Mendelian randomization: using genetic variants to assess interactions

**DOI:** 10.1093/ije/dyz161

**Published:** 2019-08-01

**Authors:** Jessica M B Rees, Christopher N Foley, Stephen Burgess

**Affiliations:** 1 Cardiovascular Epidemiology Unit, Department of Public Health and Primary Care, University of Cambridge, Cambridge, UK; 2 Edinburgh Clinical Trials Unit, Usher Institute of Population Health Sciences and Informatics, University of Edinburgh, Edinburgh, UK; 3 MRC Biostatistics Unit, University of Cambridge, Cambridge, UK

**Keywords:** Mendelian randomization, instrumental variables, interaction, causal inference, factorial randomized trial

## Abstract

**Background:**

Factorial Mendelian randomization is the use of genetic variants to answer questions about interactions. Although the approach has been used in applied investigations, little methodological advice is available on how to design or perform a factorial Mendelian randomization analysis. Previous analyses have employed a 2 × 2 approach, using dichotomized genetic scores to divide the population into four subgroups as in a factorial randomized trial.

**Methods:**

We describe two distinct contexts for factorial Mendelian randomization: investigating interactions between risk factors, and investigating interactions between pharmacological interventions on risk factors. We propose two-stage least squares methods using all available genetic variants and their interactions as instrumental variables, and using continuous genetic scores as instrumental variables rather than dichotomized scores. We illustrate our methods using data from UK Biobank to investigate the interaction between body mass index and alcohol consumption on systolic blood pressure.

**Results:**

Simulated and real data show that efficiency is maximized using the full set of interactions between genetic variants as instruments. In the applied example, between 4- and 10-fold improvement in efficiency is demonstrated over the 2 × 2 approach. Analyses using continuous genetic scores are more efficient than those using dichotomized scores. Efficiency is improved by finding genetic variants that divide the population at a natural break in the distribution of the risk factor, or else divide the population into more equal-sized groups.

**Conclusions:**

Previous factorial Mendelian randomization analyses may have been underpowered. Efficiency can be improved by using all genetic variants and their interactions as instrumental variables, rather than the 2 × 2 approach.

**Key Messages**

Factorial Mendelian randomization is an extension of the Mendelian randomization paradigm to answer questions about interactions.
There are two contexts in which factorial Mendelian randomization can be used: for investigating interactions between risk factors, and interactions between pharmacological interventions on risk factors.
While most applications of factorial Mendelian randomization have dichotomized the population as in a 2 × 2 factorial randomized trial, this approach is generally inefficient for detecting statistical interactions.
In the first context, efficiency is maximized by including all genetic variants and their cross-terms as instrumental variables for the two risk factors and their product term.
In the second context, efficiency is maximized by using continuous genetic scores rather than dichotomized scores.

## Introduction

Mendelian randomization is the use of genetic variants as proxies for interventions on risk factors to answer questions of cause and effect using observational data.^1^^,^^2^ Formally, Mendelian randomization can be viewed as instrumental variable (IV) analysis using genetic variants as IVs.^3^^,^^4^ Factorial Mendelian randomization is the use of genetic variants to answer questions about interactions. It does this by proposing a statistical model for the outcome as a function of the risk factors (or their genetic predictors) and a product term.

A statistical interaction is observed when the coefficient for the product term in the model is non-zero. A statistical interaction in the causal model for the outcome may represent a causal interaction, meaning that the effect of one risk factor on the outcome is dependent upon the value of the other risk factor.^5^^,^^6^ This may arise due to a functional or biological interaction, in which there is a mechanistic connection between the two risk factors in how they influence the outcome. However, a statistical interaction may also arise due to non-linearity in the effect of a risk factor, or due to effect modification, in which the effect of one risk factor varies in strata of the other. Hereafter, we take the word ‘interaction’ to mean a statistical interaction in the causal model for the outcome, without implying a functional interaction between the risk factors.

Factorial Mendelian randomization was proposed in the seminal paper on Mendelian randomization by Davey Smith and Ebrahim in 2003.^1^ The term is credited by the authors to Sheila Bird (https://en.wikipedia.org/wiki/Sheila_Bird). However, the idea was not readily taken up in applied practice. The concept was raised again by Davey Smith and Hemani in a 2014 review,^7^ who suggested that genetic predictors of obesity and alcohol consumption could be used to investigate the interaction between the two risk factors on risk of liver disease. This question was investigated for markers of liver function using data from the Copenhagen General Population Study in 2018;^8^ no evidence for an interaction was found.

In parallel to this, the term factorial Mendelian randomization has been used for analyses employing genetic variants as proxies for pharmacological interventions. Ference *et al*. performed factorial Mendelian randomization to compare the effect of lowering low density lipoprotein (LDL) cholesterol levels on the risk of coronary heart disease (CHD) with two different LDL-cholesterol lowering agents (ezetimibe and statin), and with a combination of both.^9^ Genetic variants associated with LDL-cholesterol were identified in the *NPC1L1* gene (proxies for ezetimibe), and the *HMGCR* gene (proxies for statins), and combined into separate gene scores. To mimic a 2 × 2 factorial randomized trial, the two gene scores were dichotomized to create a 2 × 2 contingency table. The gene scores were dichotomized at their median values so that the numbers of participants were balanced across the four groups. Ference has conducted similar analyses for PCSK9 inhibitors and statins,^10^ and for CETP inhibitors and statins.^11^ A similar 2 × 2 approach was used in each case, as well as in the analysis of obesity and alcohol mentioned above.^8^

In this paper, we consider various aspects relating to the conceptualization, design, analysis and interpretation of a factorial Mendelian randomization investigation. First, we demonstrate the analogy between factorial Mendelian randomization and a factorial randomized trial, we make a connection with multivariable Mendelian randomization, and we describe two contexts in which factorial Mendelian randomization may have utility: for investigating interactions between risk factors, and for investigating interactions between pharmacological interventions on risk factors. We present simulated data demonstrating that the 2 × 2 approach to analysis, while being conceptually appealing, is inefficient for detecting interactions. The same conclusion is reached in an applied investigation considering interactions between body mass index (BMI) and alcohol consumption on blood pressure using data from UK Biobank. Finally, we discuss the implications of our work for applied factorial Mendelian randomization investigations.

## Methods

### Factorial randomized trials and Mendelian randomization

**Figure 1:**
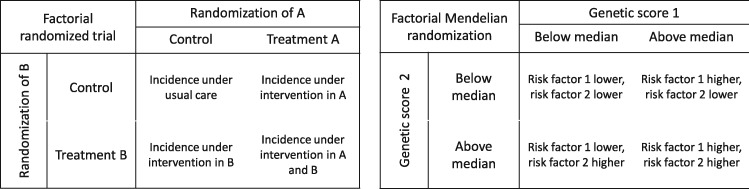
Comparison of a factorial randomized clinical trial and a factorial Mendelian randomization investigation with a 2 × 2 approach (adapted from^15^).

A factorial randomized trial allows for the simultaneous assessment of two or more treatments in a single study.^12^ In its simplest form, a 2 × 2 factorial trial investigates the effect of two binary treatments A and B on a binary outcome *Y*. Participants are randomly allocated to one of four groups: to receive treatment A only; to receive treatment B only; to receive both treatments A and B; or to receive neither treatment A nor B. The analogy between Mendelian randomization and a randomized trial has been made many times,^13^^,^^14^ and the analogy between factorial Mendelian randomization and a factorial randomized trial has also been made previously in the context of multivariable Mendelian randomization (Figure 1, adapted from^15^).

Multivariable Mendelian randomization was motivated by the problem that some genetic variants are associated with multiple risk factors, such that it is impossible to find genetic variants that are specifically associated with a particular risk factor.^15^ For illustration, we assume there are two risk factors (*X*_1_ and *X*_2_), and fit a model for the outcome in terms of the risk factors:

(1) $$E(Y|X_{1},X_{2})=\theta_{0}+\theta_{1}X_{1}+\theta_{2}X_{2}.$$

We assume that we have genetic variants *G* that satisfy the multivariable IV assumptions for risk factors *X*_1_ and *X*_2._^15^ Specifically:

- Each variant is associated with at least one of the risk factors.
- Each risk factor is associated with at least one of the genetic variants.
- Variants are not confounded in their associations with the outcome.
- Variants are not associated with the outcome conditional on the risk factors and confounders.

If we have at least two genetic variants that are valid multivariable IVs for *X*_1_ and *X*_2_, then causal effects *θ*_1_ and *θ*_2_ can be estimated from the two-stage least squares method by first regressing the risk factors on the genetic variants, and then regressing the outcome on the fitted values of the risk factors from the first-stage regressions.^16^ If summarized data on the genetic associations with the outcome (${\hat{\beta }}_{Y}$) and the risk factors (${\hat{\beta }}_{X1},\;{\hat{\beta }}_{X2}$) are available, then the same estimates can be obtained by weighted linear regression of the beta-coefficients with the intercept set to zero:

(2) $$E({\hat{\beta }}_{Y}|{\hat{\beta }}_{X1},{\hat{\beta }}_{X2})=\theta_{1}{\hat{\beta }}_{X1}+\theta_{2}{\hat{\beta }}_{X2},$$

where weights are the reciprocals of variances of the gene–outcome associations $\text{se}{\left({\hat{\beta }}_{Y}\right)}^{-2}$.^17^

In the language of a factorial randomized trial, this is referred to as an analysis performed ‘at the margins’.^18^ Estimates represent the average direct effect of each of the risk factors.^19^ If there is an interaction between the risk factors, then these are marginal estimates—they are averaged over the distribution of the other risk factor.

We can extend multivariable Mendelian randomization by adding a term to the outcome model to estimate an interaction between the risk factors:

(3) $$E(Y|X_{1},X_{2})=\theta_{0}+\theta_{1}X_{1}+\theta_{2}X_{2}+\theta_{12}X_{12}$$

where *X*_12_ is the product $X_{1}\times X_{2}$, and *θ*_12_ is the interaction effect on an additive scale. In a factorial randomized trial, this is referred to as an analysis performed ‘inside the table’, as in a 2 × 2 setting, the interaction can be estimated from the 2 × 2 contingency table.^20^ A factorial Mendelian randomization analysis is primarily interested in assessing the presence of, and estimating the interaction effect *θ*_12_.

For simplicity, we initially assume that the associations of the genetic variants with the risk factors are homogeneous in the population and do not vary with time, also that the model relating the risk factors to the outcome is correctly specified, and the effects of the risk factors (and their product) on the outcome are also homogeneous in the population and do not vary with time. We return to the question of how to interpret estimates in this and in more realistic scenarios in the Discussion.

## Two contexts: interactions between risk factors and interactions between interventions

Factorial Mendelian randomization study has been considered in two broad scenarios: (a) to estimate interaction effects between risk factors by using genetic variants as predictors of the risk factors; and (b) to identify interactions between interventions by using genetic variants as proxies for specific treatments. In the first case, the aim is to identify an interaction in the effect of two distinct risk factors on the outcome. In the second case, there may not even be two distinct risk factors (as in the example of two LDL-cholesterol lowering interventions discussed by Ference *et al*.^9^) and the aim is to identify an interaction in the associations of the genetic variants with the outcome. In this case, an interaction is inferred between the interventions for which the genetic variants are proxies. We consider these two scenarios in turn.

## Interactions between risk factors

**Figure 2:**
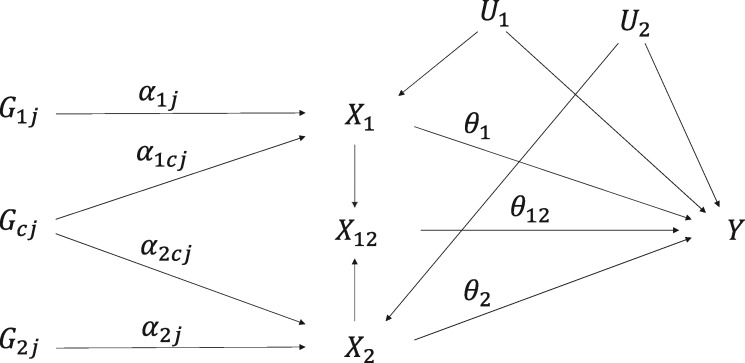
Causal directed acyclic graph illustrating relationships between variables in a factorial Mendelian randomization framework for two risk factors (*X*_1_ and *X*_2_). There are three sets of genetic variants: *G*_1_ (affecting *X*_1_ only), *G*_2_ (affecting *X*_2_ only) and *G_c_* (shared variants, affecting *X*_1_ and *X*_2_). *X*_12_ represents the product $X_{1}\times X_{2}$. The main effects of the risk factors *X*_1_ and *X*_2_ on the outcome *Y* are *θ*_1_ and *θ*_2_, and the interaction effect of *X*_1_ and *X*_2_ on *Y* is *θ*_12_. *U*_1_ and *U*_2_ are sets of confounders.

The multivariable IV assumptions imply that there is no effect of the genetic variants on the outcome except potentially indirectly via one or both of the risk factors. We divide the genetic variants into three groups: *G*_1_ contains variants that are associated with *X*_1_, *G*_2_ contains variants that are associated with *X*_2_, and *G_c_* contains shared variants that are associated with *X*_1_ and *X*_2_ (Figure 2). We can now perform two-stage least squares by first regressing the risk factors *X*_1_, *X*_2_, and the product *X*_12_ on the genetic variants, and then regressing the outcome on the fitted values of these risk factors. This analysis treats *X*_12_ as if it is a separate risk factor unrelated to *X*_1_ and *X*_2._^21^ For the second-stage regression model to be identified, at least three IVs are required, as three parameters are estimated, and all risk factors (*X*_1_, *X*_2_, *X*_12_) must be associated with at least one IV.

If we assume that the risk factors *X*_1_ and *X*_2_ are linear in the genetic variants:

(4) $$\begin{array}{c} E[X_{1}|G]=\alpha_{01}+\sum \alpha_{1j}G_{1j}+\sum \alpha_{1cj}G_{\mathrm{cj}}\;\text{and}\;\\ E[X_{2}|G]=\alpha_{02}+\sum \alpha_{2j}G_{2j}+\sum \alpha_{2cj}G_{\mathrm{cj}}, \end{array}$$

then an interaction between the risk factors means that the statistical model for the outcome includes cross-terms between the genetic variants (such as $G_{11}\times G_{21}$).^22^ This motivates the use of cross-terms between the genetic variants as separate IVs.

If all the genetic variants and their cross-terms are used as IVs, then under the homogeneity assumptions, the fitted values of the risk factors and their product term can be consistently estimated, and hence the regression model for the outcome on these fitted values (as in the two-stage least squares method) will be correctly specified. Thus the homogeneity assumptions lead to consistent estimates of the parameters in equation (3).

## Simulation study 1: interactions between risk factors

To investigate the performance of methods for estimating interactions between risk factors, we conduct a simulation study. We assume there are 10 genetic variants that are associated with *X*_1_ and 10 genetic variants that are associated with *X*_2_, and vary the number of shared variants that are associated with both *X*_1_ and *X*_2_ from 0 (20 distinct genetic variants, each associated with one risk factor) to 10 (all 10 genetic variants associated with both risk factors). All genetic variants are simulated as independent (i.e. not in linkage disequilibrium). We compare four methods:

Method 1. Full set of interactions: we consider as IVs all the genetic variants and all cross-terms—so when there are 3 shared variants, there are 114 IVs in total: 7 + 7 + 3 = 17 linear terms, 3 quadratic terms (shared variants only), 3 shared × shared variant cross-terms, 42 shared × non-shared variant cross-terms, and 49 non-shared × non-shared variant cross-terms.

Method 2. Reduced set of interactions: we consider as IVs all the genetic variants and all cross-terms between non-shared variants—so when there are 3 shared variants, there are 17 linear terms and 49 cross-terms.

Method 3. Continuous gene scores: we construct weighted gene scores for each risk factor using external weights, and take the two gene scores and their product as IVs.

Method 4. Dichotomized gene scores: we dichotomize both gene scores at their median, and take the two dichotomized gene scores and their product as IVs. This is equivalent to a 2 × 2 analysis.

The data-generating model for the simulation study is provided in the Supplementary Material, available as Supplementary data at *IJE* online. Data were generated 10 000 times for each set of parameters on 10 000 individuals. Parameters were set such that the set of genetic variants explains around 10% of the variance in each risk factor. The effect of *X*_1_ on the outcome was $\theta_{1}=0.3$, the effect of *X*_2_ on the outcome was $\theta_{2}=0.2$, and the interaction effect of *X*_12_ on the outcome took values $\theta_{12}=0.1$, 0.3, and 0.5.

## Simulation study 2: interactions between interventions

We performed a further simulation study to investigate methods for detecting interactions between interventions. We assume there are 3 independent genetic variants that are proxies for intervention A, and the same for intervention B. Fewer variants are considered here as typically variants for such an analysis will come from a single gene region for each intervention.^9^ We compare two approaches.

- Continuous gene scores: we construct weighted gene scores for changes in the risk factor corresponding to each intervention using external weights, and take the two gene scores and their product as IVs.
- Dichotomized gene scores: we dichotomize both gene scores at their median, and take the two dichotomized gene scores and their product as IVs. This is equivalent to a 2 × 2 analysis.

In each case, we regressed the outcome on the IVs, and estimated an interaction term between the gene scores that act as proxies for the interventions. As before, the data-generating model for the simulation study is provided in the Supplementary Material, available as Supplementary data at *IJE* online. Data were generated 10 000 times for each set of parameters on 10 000 individuals. The interaction effect took values 0.1, 0.3, and 0.5. We varied the minor allele frequencies of the genetic variants used as proxies for interventions A and B, drawing from a uniform distribution between 0.1 and 0.2 (uncommon), or between 0.4 and 0.5 (common), and the proportion of variance explained by the genetic variants (3, 5 or 7%).

### Applied example: the effects of BMI and alcohol on systolic blood pressure

Increased systolic blood pressure (SBP) is associated with a range of health conditions, including cardiovascular disease and diabetes.^23^^,^^24^ Whereas there have been numerous studies highlighting the adverse effects of increased BMI on SBP,^25^^,^^26^ and the adverse effects of increased alcohol consumption,^27^ there has been little research on the combined effect of BMI and alcohol consumption on SBP. We illustrate factorial Mendelian randomization by performing an analysis using individual participant data from UK Biobank to estimate the interaction effect of BMI and alcohol consumption on SBP. UK Biobank is a prospective, population-based cohort consisting of ∼500 000 participants aged from 40 to 69 years at baseline living in the UK. For the analysis, we considered 291 781 unrelated participants of European descent who passed data quality control measures and had genetic data available.

We used the 77 genome-wide significant variants from a meta-analysis by the Genetic Investigation of ANthropometric Traits (GIANT) consortium in participants of European ancestry to act as IVs for BMI.^28^ For alcohol, we identified 10 genetic variants in the *ADH1B* gene region that have been shown to be associated with alcohol consumption.^29^ We performed factorial Mendelian randomization analyses using the full set of interactions, continuous gene scores, and dichotomized gene scores. We also performed analyses separately using the lead variant from the *ADH1B* gene region (rs1229984) as the sole IV for alcohol consumption, as was done in the analysis by Carter *et al*.^8^

## Results

### Simulation study 1: interactions between risk factors

Results from the simulation study for estimating interactions between risk factors are displayed in Table 1 (no shared variants) and Table 2 (varying the number of shared variants). All four approaches provided unbiased estimates of the interaction effect in all scenarios, with coverage for the 95% confidence interval close to the nominal 95% level. Power varied considerably between the methods. With no shared variants, method 1 (full set of interactions) and method 2 (reduced set of interactions) are equivalent and gave the most efficient estimates throughout. Method 3 (continuous gene scores) was less efficient, and method 4 (dichotomized gene scores) was the least efficient. With shared variants, method 1 was the most efficient throughout, and its efficiency was not strongly affected by the risk factors having genetic predictors in common. Between methods 2 and 3, method 2 was more efficient when most of the variants were non-shared, whereas method 3 was more efficient when most of the variants were shared. Again, method 4 was the least efficient in all scenarios. This suggests that the 2 × 2 approach may be underpowered in practice, and instead approaches using all genetic variants and their cross-terms should be considered.

**Table 1.** Simulation study results for interactions between risk factors with no shared variants: median estimate, standard deviation (SD) of estimates, median standard error (SE), empirical power (%) to reject null at 5% significance, and empirical coverage (%) of 95% confidence interval

	Median	SD	Median SE	Power (%)	Coverage (%)
Methods 1 and 2—full set of interactions:^a^					
$\theta_{1}=0.3$	0.3013	0.0917	0.0910	90.2	95.0
$\theta_{2}=0.2$	0.2022	0.0952	0.0945	57.1	94.9
$\theta_{12}=0.1$	0.1101	0.0721	0.0718	33.7	94.6
$\theta_{1}=0.3$	0.3043	0.0918	0.0910	91.0	95.0
$\theta_{2}=0.2$	0.2034	0.0947	0.0945	57.9	95.5
$\theta_{12}=0.3$	0.3080	0.0722	0.0718	98.8	95.2
$\theta_{1}=0.3$	0.3048	0.0911	0.0909	90.7	95.2
$\theta_{2}=0.2$	0.2050	0.0944	0.0945	58.4	95.2
$\theta_{12}=0.5$	0.5073	0.0715	0.0718	100.0	95.2
Method 3—continuous gene scores:					
$\theta_{1}=0.3$	0.2993	0.1362	0.1333	61.4	95.4
$\theta_{2}=0.2$	0.1991	0.1415	0.1386	30.9	95.5
$\theta_{12}=0.1$	0.1010	0.1113	0.1091	15.4	95.5
$\theta_{1}=0.3$	0.2998	0.1359	0.1332	61.9	95.6
$\theta_{2}=0.2$	0.2019	0.1405	0.1387	31.5	95.8
$\theta_{12}=0.3$	0.3000	0.1106	0.1091	77.5	95.8
$\theta_{1}=0.3$	0.3004	0.1352	0.1331	61.5	95.4
$\theta_{2}=0.2$	0.2008	0.1409	0.1385	30.7	95.6
$\theta_{12}=0.5$	0.4995	0.1107	0.1092	98.7	95.6
Method 4—dichotomized gene scores:					
$\theta_{1}=0.3$	0.2986	0.2155	0.2072	31.0	95.7
$\theta_{2}=0.2$	0.1989	0.2246	0.2168	15.0	96.2
$\theta_{12}=0.1$	0.1022	0.1786	0.1720	8.0	95.9
$\theta_{1}=0.3$	0.3039	0.2145	0.2074	32.1	95.8
$\theta_{2}=0.2$	0.2047	0.2236	0.2164	15.2	96.2
$\theta_{12}=0.3$	0.2972	0.1777	0.1722	41.8	96.0
$\theta_{1}=0.3$	0.3010	0.2148	0.2073	31.4	96.2
$\theta_{2}=0.2$	0.2002	0.2233	0.2163	15.3	96.1
$\theta_{12}=0.5$	0.5002	0.1776	0.1718	80.7	96.1

a As there are no shared variants, methods 1 and 2 are equivalent.

**Table 2.** Simulation study results for interaction term between risk factors varying number of shared variants: median estimate of $\theta_{12}=0.3$, standard deviation (SD) of estimates, median standard error (SE), empirical power (%) to reject null at 5% significance, and empirical coverage (%) of 95% confidence interval

Shared variants	Total IVs	Median	SD	Median SE	Power (%)	Coverage (%)
Method 1—full set of interactions:						
0^a^	120	0.3080	0.0722	0.0718	98.8	95.2
1	119	0.3080	0.0723	0.0719	98.8	95.0
3	114	0.3090	0.0717	0.0716	98.9	95.3
5	105	0.3078	0.0716	0.0707	98.9	94.9
8	84	0.3073	0.0682	0.0687	99.3	95.2
10	65	0.3056	0.0670	0.0673	99.2	95.3
Method 2—reduced set of interactions:						
1	100	0.3073	0.0804	0.0794	96.7	94.9
3	66	0.3088	0.1003	0.0997	86.1	95.2
5	40	0.3056	0.1340	0.1334	63.2	95.7
8	16	0.3054	0.2520	0.2471	23.9	97.1
10	10	0.3057	0.3883	0.3891	8.7	99.3
Method 3—continuous gene scores:						
0	3	0.3000	0.1106	0.1091	77.5	95.8
1	3	0.3005	0.1111	0.1088	77.8	95.4
3	3	0.2998	0.1051	0.1048	81.0	95.6
5	3	0.3015	0.0997	0.0980	85.6	95.5
8	3	0.3003	0.0857	0.0858	93.0	95.8
10	3	0.2993	32.31	0.1711	42.7	99.2
Method 4—dichotomized gene scores:						
0	3	0.2972	0.1777	0.1722	41.8	96.0
1	3	0.3028	0.1757	0.1724	42.2	96.3
3	3	0.3002	0.1818	0.1773	39.8	96.4
5	3	0.3005	0.1948	0.1884	36.6	96.6
8	3	0.3007	0.2474	0.2340	25.7	97.2
10	3	0.2896	133.5	1.3578	0.7	100.0

a When there are no shared variants, methods 1 and 2 are equivalent.

We also varied the strength of the genetic variants due to potential concerns about weak instruments.^30^ We considered scenarios in which the genetic variants explained 1% and 5% of variance in the risk factors. Although substantial weak instrument bias was observed for the main effects, no bias was observed for the interaction term, even when there were 100 IVs in the analysis and F-statistics and conditional F-statistics^31^ for the product term were ∼1 (Supplementary Tables A1 and A2, available as Supplementary data at *IJE* online). Similar findings were observed in a one-sample setting when varying the direction of confounder effects on the risk factor and outcome (results not shown). We also performed the simulation study centering the values of the risk factors to reduce the impact of collinearity. This changed the mean estimates of the main effects *θ*_1_ and *θ*_2_ and improved precision for the main effect estimates, but estimates and inferences for the interaction term *θ*_12_ were unchanged (Supplementary Table A3, available as Supplementary data at *IJE* online). These additional simulations suggest that factorial Mendelian randomization should only be used when the interaction is the main object of interest, and numerical estimates for the main effects from this model should be interpreted with caution.

### Simulation study 2: interactions between interventions

Results from the simulation study for estimating interactions between the gene scores that act as proxies for the interventions are displayed in Table 3. Whereas the numerical values of estimates differed between the two approaches, a consistent finding was that power to detect an interaction was greater using continuous gene scores than using dichotomized gene scores. Varying the proportion of variance explained by the genetic variants had no discernable effect on the power to detect an interaction. This can be seen by comparing scenarios 1, 2 and 3, and scenarios 5 and 6. However, varying the minor allele frequency had a strong effect on power, with greater power when the minor allele frequency was close to 0.5. This can be seen by comparing scenarios 2, 4 and 5, and scenarios 3 and 6. This suggests that ensuring comparable size between subgroups is an important factor for efficient detection of interactions, and can be more important than ensuring that the strongest variant is used in the analysis.

**Table 3.** Simulation study results for interaction between interventions: median estimate, standard deviation (SD) of estimates, median standard error (SE), and empirical power (%) to reject null at 5% significance. The minor allele frequencies and proportion of variance explained for variants that are proxies for interventions A and B are varied between scenarios

	Continuous gene scores				Dichotomized gene scores			
	Median	SD	Median SE	Power	Median	SD	Median SE	Power
Scenario 1: (A) common variants, 3%; (B) common variants, 3%								
θ_12_=0.1	0.0583	0.0420	0.0417	29.3	0.0368	0.0423	0.0421	13.5
θ_12_=0.3	0.0330	0.0080	0.0078	98.7	0.1102	0.0429	0.0423	73.5
θ_12_=0.5	0.0224	0.0034	0.0032	100.0	0.1846	0.0428	0.0427	98.9
Scenario 2: (A) common variants, 5%; (B) common variants, 5%								
θ_12_=0.1	0.0484	0.0343	0.0343	29.1	0.0372	0.0420	0.0422	13.5
θ_12_=0.3	0.0304	0.0074	0.0072	98.8	0.1108	0.0424	0.0423	74.3
θ_12_=0.5	0.0212	0.0033	0.0030	100.0	0.1851	0.0439	0.0427	99.0
Scenario 3: (A) common variants, 3%; (B) common variants, 7%								
θ_12_=0.1	0.0498	0.0350	0.0352	29.2	0.0371	0.0422	0.0422	14.1
θ_12_=0.3	0.0305	0.0075	0.0072	99.0	0.1106	0.0426	0.0423	74.2
θ_12_=0.5	0.0213	0.0033	0.0030	100.0	0.1844	0.0430	0.0427	99.1
Scenario 4: (A) uncommon variants, 5%; (B) uncommon variants, 5%								
θ_12_=0.1	0.0824	0.1152	0.1150	10.9	0.0168	0.0435	0.0430	7.0
θ_12_=0.3	0.1082	0.0519	0.0500	58.8	0.0526	0.0434	0.0430	23.3
θ_12_=0.5	0.0996	0.0300	0.0278	94.6	0.0879	0.0436	0.0430	53.0
Scenario 5: (A) common variants, 5%; (B) uncommon variants, 5%								
θ_12_=0.1	0.0669	0.0699	0.0685	16.7	0.0246	0.0434	0.0425	9.1
θ_12_=0.3	0.0618	0.0211	0.0204	85.5	0.0763	0.0433	0.0426	42.8
θ_12_=0.5	0.0489	0.0109	0.0097	99.9	0.1279	0.0434	0.0428	84.1
Scenario 6: (A) common variants, 3%; (B) uncommon variants, 7%								
θ_12_=0.1	0.0748	0.0756	0.0742	17.8	0.0259	0.0432	0.0426	9.7
θ_12_=0.3	0.0649	0.0221	0.0215	85.4	0.0758	0.0430	0.0426	42.9
θ_12_=0.5	0.0510	0.0113	0.0101	99.9	0.1271	0.0435	0.0428	83.9

### Applied example: the effects of BMI and alcohol on systolic blood pressure

The lead variant (rs1229984) explained 0.24% of the variance in alcohol consumption, whereas the 10 variants explained 0.28% of the variance. Although the alcohol-decreasing allele of the rs1229984 variant is dominant, its frequency is only 2.5%. Dichotomizing participants based on this variant led to unequal groups in the population, whereas dichotomizing based on the 10 variant score led to equal groups (Table 4). However, the difference in mean alcohol levels between subgroups was reduced when using the 10 variant score, as most of the difference is due to the rs1229984 variant.

**Table 4.** Subgroups defined by genetic predictors of BMI and alcohol consumption: numbers (%) of participants and mean (standard deviation) of body mass index, alcohol consumption and systolic blood pressure in 2 × 2 subgroups when either 10 genetic variants or the rs1229984 variant used as IVs for alcohol consumption

		Mean (SD)		
	Participants (%)	BMI (kg/m^2^)	Alcohol (units/day)	SBP (mmHg)
Overall	291, 781 (100.0)	27.1 (4.51)	2.54 (2.58)	140.0 (19.8)
10 variants for alcohol:				
Low BMI, low alcohol	73, 003 (25.0)	26.6 (4.25)	2.50 (2.52)	140.6 (20.6)
High BMI, low alcohol	72, 889 (25.0)	27.5 (4.65)	2.47 (2.50)	141.2 (20.6)
Low BMI, high alcohol	72, 888 (25.0)	26.7 (4.30)	2.61 (2.68)	140.8 (20.7)
High BMI, high alcohol	73, 001 (25.0)	27.6 (4.71)	2.59 (2.59)	141.3 (20.6)
rs1229984 variant for alcohol:				
Low BMI, low alcohol	6, 997 (2.4)	26.3 (4.10)	2.00 (2.04)	139.2 (20.2)
High BMI, low alcohol	6, 863 (2.4)	27.3 (4.50)	1.95 (1.99)	139.7 (20.2)
Low BMI, high alcohol	138, 894 (47.6)	26.7 (4.28)	2.59 (2.59)	140.8 (20.6)
High BMI, high alcohol	139, 027 (47.6)	27.6 (4.69)	2.56 (2.56)	141.3 (20.6)

Estimates of the interaction between BMI and alcohol consumption are displayed in Table 5. For the dichotomized gene scores, efficiency is greater when the rs1229984 variant is used, suggesting the importance of dichotomizing the risk factor at a natural break in its distribution (if one exists) rather than ensuring that subgroups are equal in size. However, efficiency is strikingly improved using the full set of interactions, with the standard error decreasing over 10-fold using the 10 variants, and by a factor of 4 using the rs1229984 variant, compared with the 2 × 2 analysis. All estimates are compatible with the null, suggesting a lack of interaction in the effects of BMI and alcohol on SBP. There was no evidence of weak instrument bias, even though up to 857 IVs were used in the analyses and F-statistics were generally low (Supplementary Table A4, available as Supplementary data at *IJE* online).

**Table 5.** Factorial Mendelian randomization results for applied example: estimates of interaction between BMI and alcohol consumption on systolic blood pressure; estimates are in mmHg units per 1 kg/m^2^ change in BMI and 1 unit/day change in alcohol consumption

	Total IVs	Estimate	Standard error	*P*-value
10 variants for alcohol				
Method 1: full set of interactions	857	0.0023	0.0503	0.96
Method 2: continuous gene scores	3	0.0655	0.3402	0.85
Method 3: binary gene scores	3	0.1011	0.6411	0.87
rs1229984 variant for alcohol				
Method 1: full set of interactions	149	−0.0170	0.1136	0.88
Method 2: continuous gene scores	3	0.1917	0.3725	0.61
Method 3: binary gene scores	3	0.1499	0.4174	0.72

## Discussion

In this paper, we have provided a brief review of factorial Mendelian randomization, an approach that uses genetic variants as IVs to detect interactions. We have described two broad scenarios in which factorial Mendelian randomization has been implemented: to explore interactions between risk factors, and to explore interactions between interventions. Although most (perhaps even all) factorial Mendelian randomization analyses have been conducted using a 2 × 2 approach in which the sample is divided into four subgroups, we have shown that this approach is generally inefficient, particularly for exploring interactions between risk factors. This has been demonstrated in simulation studies, and in an applied example in which a 4- to 10-fold improvement in efficiency was observed by an analysis using the full set of interactions between the genetic variants as IVs.

### Choice of variants

Our findings suggest that factorial Mendelian randomization analyses should be conducted using all available genetic variants that are valid instruments, i.e. that satisfy the multivariable IV assumptions. Analyses should not only include the genetic variants as main effects, but also all relevant two-way cross-terms. A similar conclusion was made in a different context by Bollen and Paxton.^22^ If investigators want to perform a 2 × 2 analysis, this should be done to illustrate the method rather than being the main analysis for testing the presence of an interaction. For a 2 × 2 analysis, the primary consideration for choosing genetic variants should be to divide the population at a natural break in the distribution of the risk factor, in order to maximize the difference between the mean level of the risk factor in the two halves of the population. If there is no natural break in the distribution, then investigators should find a division that splits the population as far as possible into equal groups. This may entail selecting genetic variants that explain less variance in the risk factor, but have minor allele frequency closer to 50%. There can also be substantial benefit in including multiple variants in a single gene region in an analysis, even if these variants only explain a small additional proportion of variance in the risk factor.

### Weak instrument bias and efficiency

Conventionally, it is discouraged to use large numbers of genetic variants that are not strongly associated with the risk factor in a Mendelian randomization analysis due to weak instrument bias.^32^ Although we did not detect any bias from weak instruments on interaction terms in our simulations, we acknowledge that users of the method may be reluctant to use hundreds of cross-terms as IVs. We would therefore encourage the use of continuous gene score methods as sensitivity analyses. Such analyses estimate fewer parameters, so should be less susceptible to bias. However, this advice is precautionary; no evidence of weak instrument bias in interaction estimates was observed in our simulations.

### Summarized data

Whereas multivariable Mendelian randomization can be performed using summarized data that are typically reported from genome-wide association studies by large consortia, this is not possible for factorial Mendelian randomization. If summarized association estimates are available on genetic associations with the product of the two risk factors, as well as associations with the risk factors individually, then the interaction effect can in principle be estimated by weighted linear regression of the beta-coefficients as in multivariable Mendelian randomization. However, if association estimates are only available for genetic variants, then the regression model is not identified asymptotically due to collinearity, and finite-sample estimates will be biased.^33^ Association estimates for some cross-terms of genetic variants are additionally required. Hence, factorial Mendelian randomization can be performed using summarized data, but only if bespoke summarized data are available on associations of genetic variants and their cross-terms with the risk factors and their product.

### Interpretation of the interaction effect

If genetic variants each satisfy the assumptions of an IV, then an interaction between risk factors has a causal interpretation. If the two risk factors are associated with the outcome then an interaction will exist on at least one of the additive or multiplicative scales.^6^ However, there is no way of distinguishing a purely statistical interaction from a mechanistic or biological interaction based on observational data. We therefore advise caution in the interpretation of interaction findings, as a statistical interaction can arise due to non-linearity in the effect of a risk factor, or because of the scale on which the outcome is measured (for example, an interaction may occur on the original scale, but not on a log-transformed scale). When considering an interaction between interventions, researchers can investigate whether there is an interaction between the interventions on the risk factor(s) as well as on the outcome. This may help reveal where any biological interaction may take place.

Causal estimates from IV analysis have a clear interpretation in two cases: under the monotonicity assumption, and under the homogeneity assumption.^34^ In a randomized controlled trial in which random allocation is taken as the IV and the treatment is the risk factor, monotonicity means that there are no individuals in the population (known as ‘defiers’) who would take the treatment only if they were randomly allocated to the control group, and not if they were allocated to the treatment group. Under monotonicity, all individuals are either ‘always-takers’ (they would always take the treatment whether assigned to or not), ‘never-takers’ (they would never take the treatment whether assigned to or not), or ‘compliers’ (they would take the treatment if and only if assigned to do so).^35^ Under the monotonicity assumption, the IV estimate represents the complier average causal effect—the average causal effect amongst compliers.^36^ However, these definitions suppose that the IV and risk factor are binary. In Mendelian randomization, these variables are typically continuous, and so the straightforward interpretation of an IV estimate as a single complier average causal effect is lost—it instead represents a weighted average of complier average causal effects.^37^ In contrast, the IV estimate under the homogeneity assumption represents the average causal effect. In its simplest form, the homogeneity assumption states that causal effects are identical in all individuals in the population. Weaker versions of this assumption have been proposed.

If there is a non-zero interaction between the risk factors, then the homogeneity assumption in the multivariable Mendelian randomization model is violated, and the IV estimate only has a clear interpretation under the monotonicity assumption. However, the homogeneity assumption in the factorial Mendelian randomization model may still hold, if there is homogeneity in the effects of the two risk factors and their product on the outcome. Hence under homogeneity, the interaction effect has an interpretation as an average causal effect.

A further potential complication arises if genetic associations with the risk factor or outcome vary over time. As genetic variants are assigned at conception for all individuals and tend to influence risk factor levels throughout the life-course, Mendelian randomization estimates are naturally interpreted as the impact of a life-long change in the trajectory of a risk factor.^38^ Hence the natural interpretation of an interaction effect is that of a statistical interaction in the relationship between the outcome and the risk factors that relates to long-term changes in the risk factors. If genetic associations vary over time, then the interpretation of the causal estimate from Mendelian randomization is unclear. This is true for a conventional Mendelian randomization analysis as well as for a factorial Mendelian randomization analysis. One notable case to consider is if the risk factors have mutual effects on each other, as in the case of a feedback mechanism. In this situation, provided that the associations of the genetic variants with the risk factors remain linear (which would occur if all relationships between variables are linear), then this would mean that all genetic variants are associated with both risk factors. A factorial Mendelian randomization analysis would still hold for the causal interaction between the risk factors, as in the examples with shared genetic variants described earlier in the paper. Hence feedback between the risk factors does not necessarily lead to a non-zero interaction estimate. However, if the two variables of interest have a complex longitudinal relationship, and in particular if there are mutual dependencies that might vary over time, then extra caution should be taken in interpreting results from a Mendelian randomization investigation, especially numerical estimates of causal effects. This advice is also relevant if the effects of the risk factors on the outcome may vary over time (for example if there is a critical period when exposure to the risk factor influences the outcome). If the associations between variables became non-linear, then it may be worth considering using the control function approach, an extension to the two-stage least squares method that makes stronger assumptions, but can result in more efficient estimation.^39^

## Comparison with previous work

Previous work investigating interactions using IVs has been limited. A formal framework for defining interaction effects in the context of clinical trials was proposed by Blackwell,^40^ who used the language of principal stratification (compliance classes and monotonicity) to define local average interaction effects in a similar way to how local average causal effects (also called complier-averaged causal effects) are defined for single risk factors.^41^ However, the principal stratification framework presupposes that risk factors are binary (or categorical) to assign compliance classes, whereas risk factors in Mendelian randomization are typically continuous. Additionally, the principal stratification framework presupposes a single binary IV, whereas Mendelian randomization investigations often use multiple genetic variants. There is therefore little practical advice in the literature on how to perform a factorial Mendelian randomization analysis.

## Limitations

There are several limitations to this work. We rely on the assumption that all genetic variants included in our analyses are valid IVs. The IV assumptions may be violated by including genetic variants that are associated with the outcome independently of the risk factors. This violation would result in biased estimates, and could potentially lead to incorrect inferences on the presence of an interaction effect. Our recommendations rely on simulated data. Different choices for the parameters included in the simulation studies may have resulted in different conclusions. However, our findings were robust to different choices of parameters considered in this paper, they correspond to what we know about the theoretical properties of estimators, and similar conclusions were observed from the applied analysis. We have only considered interactions on an additive scale, although interactions could be considered on a multiplicative scale by log-transforming the outcome. Finally, we have not considered the impact of model misspecification on estimates. It would not be possible to perform simulation studies corresponding to all possible ways that model misspecification could occur, meaning that our recommendations cannot be proven to be optimal in all settings. We believe that we have chosen parameters and scenarios that are relevant to modern Mendelian randomization analyses.

## Conclusion

Overall, factorial Mendelian randomization is a promising technique for assessing interactions using genetic variants as IVs. Our findings suggest that current applications of factorial Mendelian randomization based on a 2 × 2 analysis could be improved by better selection of genetic variants, and by better choice of analysis method.

## Funding

This work was supported by the UK Medical Research Council (MC_UU_00002/7) and the NIHR Cambridge Biomedical Research Centre. J.M.B.R. is supported by the British Heart Foundation (grant number FS/14/59/31282). S.B. is supported by a Sir Henry Dale Fellowship jointly funded by the Wellcome Trust and the Royal Society (Grant Number 204623/Z/16/Z). The views expressed are those of the authors and not necessarily those of the NHS, the NIHR or the Department of Health and Social Care.

## Supplementary Material

dyz161_Supplementary_Data

## References

[1] Davey Smith G, Ebrahim S. ‘ Mendelian randomization’: can genetic epidemiology contribute to understanding environmental determinants of disease? Int J Epidemiol 2003;32:1–22. 12689998 10.1093/ije/dyg070

[2] Burgess S, Thompson SG, Mendelian Randomization: methods for Using Genetic Variants in Causal Estimation. Boca Raton, FL: Chapman & Hall, 2015.

[3] Lawlor D, Harbord R, Sterne J, Timpson N, Davey Smith G. Mendelian randomization: using genes as instruments for making causal inferences in epidemiology. Statist Med 2008;27:1133–63. 10.1002/sim.3034 17886233

[4] Didelez V, Sheehan N. Mendelian randomization as an instrumental variable approach to causal inference. Stat Methods Med Res 2007;16:309–30. 17715159 10.1177/0962280206077743

[5] Cox DR. Interaction. Int Stat Rev 1984;52:1–24.

[6] VanderWeele TJ Explanation in Causal Inference: Methods for Mediation and Interaction. New York, NY: Oxford University Press; 2015.

[7] Davey Smith G, Hemani G. Mendelian randomization: genetic anchors for causal inference in epidemiological studies. Hum Mol Genet 2014;23:R89–98. 25064373 10.1093/hmg/ddu328 PMC4170722

[8] Carter AR, Borges MC, Benn M et al Combined association of body mass index and alcohol consumption with biomarkers for liver injury and incidence of liver disease: a Mendelian randomization study. JAMA Netw Open 2019;2:e190305. 30848805 10.1001/jamanetworkopen.2019.0305 PMC6484655

[9] Ference BA, Majeed F, Penumetcha R, Flack JM, Brook RD. Effect of naturally random allocation to lower low-density lipoprotein cholesterol on the risk of coronary heart disease mediated by polymorphisms in *NPC1L1*, *HMGCR*, or both: a 2 × 2 factorial Mendelian randomization study. J Am Coll Cardiol 2015;65:1552–61. 25770315 10.1016/j.jacc.2015.02.020 PMC6101243

[10] Ference BA, Robinson JG, Brook RD et al Variation in *PCSK9* and *HMGCR* and risk of cardiovascular disease and diabetes. N Engl J Med 2016;375:2144–53. 27959767 10.1056/NEJMoa1604304

[11] Ference BA, Kastelein JJ, Ginsberg HN et al Association of genetic variants related to CETP inhibitors and statins with lipoprotein levels and cardiovascular risk. JAMA 2017;318:947–56. 28846118 10.1001/jama.2017.11467 PMC5710502

[12] Stampfer MJ, Buring JE, Willett W, Rosner B, Eberlein K, Hennekens CH. The 2 × 2 factorial design: Its application to a randomized trial of aspirin and U.S. physicians. Statist Med 1985;4:111–16. 10.1002/sim.4780040202 4023472

[13] Hingorani A, Humphries S. Nature’s randomised trials. Lancet 2005;366:1906–908. 16325682 10.1016/S0140-6736(05)67767-7

[14] Swanson SA, Tiemeier H, Ikram MA, Hernán MA. Nature as a trialist?: deconstructing the analogy between Mendelian randomization and randomized trials. Epidemiology 2017;28:653–59. 28590373 10.1097/EDE.0000000000000699 PMC5552969

[15] Burgess S, Thompson SG. Multivariable Mendelian randomization: the use of pleiotropic genetic variants to estimate causal effects. Am J Epidemiol 2015;181:251–60. 25632051 10.1093/aje/kwu283 PMC4325677

[16] Sanderson E, Davey Smith G, Windmeijer F, Bowden J. An examination of multivariable Mendelian randomization in the single sample and two-sample summary data settings. Int J Epidemiol 2019:48:713–27. 10.1093/ije/dyy262 PMC6734942 30535378

[17] Burgess S, Dudbridge F, Thompson SG. Re: “Multivariable Mendelian randomization: the use of pleiotropic genetic variants to estimate causal effects”. Am J Epidemiol 2015;181:290–91. 25660081 10.1093/aje/kwv017

[18] McAlister FA, Straus SE, Sackett DL, Altman DG. Analysis and reporting of factorial trials: a systematic review. J Am Med Assoc 2003;289:2545–53. 10.1001/jama.289.19.2545 12759326

[19] Burgess S, Thompson DJ, Rees JM, Day FR, Perry JR, Ong KK. Dissecting causal pathways using Mendelian randomization with summarized genetic data: application to age at menarche and risk of breast cancer. Genetics 2017;207:481–7. 28835472 10.1534/genetics.117.300191 PMC5629317

[20] Dakin H, Gray A. Economic evaluation of factorial randomised controlled trials: challenges, methods and recommendations. Statist Med 2017;36:2814–30. 10.1002/sim.7322 PMC5599939 28470760

[21] Wooldridge JM Econometric Analysis of Cross Section and Panel Data. Chapter 18: Estimating Average Treatment Effects. Cambridge, MA: MIT Press; 2002.

[22] Bollen KA, Paxton P, Two-stage least squares estimation of interaction effects In: Schumacker RE, Marcoulides GA (eds). Interaction and Nonlinear Effects in Structural Equation Modeling. New Jersey: Lawrence Erlbaum Associates Publishers; 1998, pp.125–51.

[23] Lewington S, Clarke R, Qizilbash N, Peto R, Collins R. Age-specific relevance of usual blood pressure to vascular mortality: a meta-analysis of individual data for one million adults in 61 prospective studies. Lancet 2002;360:1903–13. 12493255 10.1016/s0140-6736(02)11911-8

[24] Wei GS, Coady SA, Goff DC et al Blood pressure and the risk of developing diabetes in african americans and whites: ARIC, CARDIA, and the Framingham Heart Study. Diabetes Care 2011;34:873–79. 21346180 10.2337/dc10-1786 PMC3064044

[25] Droyvold WB, Midthjell K, Nilsen TI, Holmen J. Change in body mass index and its impact on blood pressure: a prospective population study. Int J Obes 2005;29:650–55. 10.1038/sj.ijo.0802944 15809666

[26] Gelber RP, Gaziano JM, Manson JE, Buring JE, Sesso HD. A prospective study of body mass index and the risk of developing hypertension in men. Am J Hypertens 2007;20:370–77. 17386342 10.1016/j.amjhyper.2006.10.011 PMC1920107

[27] Roerecke M, Kaczorowski J, Tobe SW, Gmel G, Hasan OSM, Rehm J. The effect of a reduction in alcohol consumption on blood pressure: a systematic review and meta-analysis. Lancet Public Health 2017;2:e108–20. 29253389 10.1016/S2468-2667(17)30003-8 PMC6118407

[28] Locke AE, Kahali B, Berndt SI et al Genetic studies of body mass index yield new insights for obesity biology. Nature 2015;518:197–206. 25673413 10.1038/nature14177 PMC4382211

[29] Lewis SJ, Zuccolo L, Smith GD et al Fetal alcohol exposure and IQ at age 8: evidence from a population-based birth-cohort study. PLOS One 2012;7:e49407. 23166662 10.1371/journal.pone.0049407 PMC3498109

[30] Burgess S, Thompson SG. Bias in causal estimates from Mendelian randomization studies with weak instruments. Statist Med 2011;30:1312–23. 10.1002/sim.4197 21432888

[31] Sanderson E, Windmeijer F. A weak instrument F-test in linear IV models with multiple endogenous variables. J Econ 2016;190:212–21. 10.1016/j.jeconom.2015.06.004 PMC5669336 29129953

[32] Burgess S, Thompson SG, CRP CHD Genetics Collaboration. Avoiding bias from weak instruments in Mendelian randomization studies. Int J Epidemiol 2011;40:755–64. 21414999 10.1093/ije/dyr036

[33] Rees JMB. Robust Methods in Mendelian Randomization. Chapter 5: Extending Mendelian Randomization to a Factorial Framework to Detect Interaction Effects. Cambridge, UK: University of Cambridge; 2019.

[34] Hernán MA, Robins JM. Instruments for causal inference: an epidemiologist’s dream? Epidemiology 2006;17:360–72. 16755261 10.1097/01.ede.0000222409.00878.37

[35] Frangakis CE, Rubin DB. Principal stratification in causal inference. Biometrics 2002;58:21–29. 11890317 10.1111/j.0006-341x.2002.00021.x PMC4137767

[36] Yau LHY, Little RJ. Inference for the complier-average causal effect from longitudinal data subject to noncompliance and missing data, with application to a job training assessment for the unemployed. J Am Stat Assoc 2001;96:1232–44.

[37] Angrist JD, Graddy K, Imbens GW. The interpretation of instrumental variables estimators in simultaneous equations models with an application to the demand for fish. Rev Econ Studies 2000;67:499–527.

[38] Labrecque JA, Swanson SA. Interpretation and potential biases of Mendelian randomization estimates with time-varying exposures. Am J Epidemiol 2019;188:231–38. 30239571 10.1093/aje/kwy204

[39] Guo Z, Small DS. Control function instrumental variable estimation of nonlinear causal effect models. J Mach Learn Res 2016;17:3448–82.

[40] Blackwell M. Instrumental variable methods for conditional effects and causal interaction in voter mobilization experiments. J Am Stat Assoc 2017;112:590–99.

[41] Imbens GW, Angrist JD. Identification and estimation of local average treatment effects. Econometrica 1994;62:467–75.

