# Supplementary material for: Factorial Mendelian randomization: using genetic variants to assess interactions
Source: Int J Epidemiol. 2019 Aug 1;49(4):1147–58. doi: 10.1093/ije/dyz161 (PMC7750987; doi:10.1093/ije/dyz161)
Supplement: dyz161_Supplementary_Data [file dyz161_supplementary_data.pdf]

Supplementary Material

In the Supplementary Material, we provide more detail on the two simulation studies and the applied example presented in the paper.

Simulation study 1: interactions between risk factors

The two risk factors X_1 and X_2 were generated for $i = 1, 2, \ldots, 10\,000$ participants from the following data-generating model:

$$X_{1i} = \sum_{j=1}^{J_1} \alpha_{1j} G_{1ji} + \sum_{j=1}^{J_c} \alpha_{1cj} G_{cji} + U_{1i} + \epsilon_{1i} \quad \text{and}$$
$$X_{2i} = \sum_{j=1}^{J_2} \alpha_{2j} G_{2ji} + \sum_{j=1}^{J_c} \alpha_{2cj} G_{cji} + U_{2i} + \epsilon_{2i}\,,$$

where $\boldsymbol{G_1}$ and $\boldsymbol{G_2}$ are the genetic variants associated with X_1 and X_2 respectively, and $\boldsymbol{G_c}$ are the set of shared variants that are associated with both X_1 and X_2 (bold font represents vectors). The genotypes (0, 1 or 2) were generated independently from binomial distributions $\text{Bin}(2, MAF_j)$, where MAF_j represents the minor allele frequency (MAF) of the jth genetic variant, and was drawn from a uniform distribution $\text{Unif}(0.1, 0.5)$. $\boldsymbol{\alpha_1}$ and $\boldsymbol{\alpha_{1_c}}$ represent the effects of the genetic variants $\boldsymbol{G_1}$ and $\boldsymbol{G_c}$ on X_1, and $\boldsymbol{\alpha_2}$ and $\boldsymbol{\alpha_{2_c}}$ represent the effects of the genetic variants $\boldsymbol{G_2}$ and $\boldsymbol{G_c}$ on X_2. The genetic associations were calculated so that $\boldsymbol{G_1}$ and $\boldsymbol{G_c}$, and $\boldsymbol{G_2}$ and $\boldsymbol{G_c}$, explained $\sigma_1^2 = \sigma_2^2 = 10\%$ of the variance in X_1 and X_2 respectively. To ensure that each genetic variant explained the same amount of variation in the risk factor, we rearranged:

$$\text{var}(G_{1j}) = \sigma_1^2 = 2 \times \alpha_{1j}^2 MAF_{1j}(1 - MAF_{1j}) \quad \text{and}$$
$$\text{var}(G_{2j}) = \sigma_2^2 = 2 \times \alpha_{2j}^2 MAF_{2j}(1 - MAF_{2j})\,,$$

to calculate the genetic associations:

$$\alpha_{1j} = \sqrt{\frac{\sigma_1^2/(J_1 + J_c)}{2 \times MAF_{1j}(1 - MAF_{1j})}}\,,$$
$$\alpha_{1cj} = \sqrt{\frac{\sigma_1^2/(J_1 + J_c)}{2 \times MAF_{cj}(1 - MAF_{cj})}}\,,$$
$$\alpha_{2j} = \sqrt{\frac{\sigma_2^2/(J_1 + J_c)}{2 \times MAF_{2j}(1 - MAF_{2j})}}\,,$$
$$\alpha_{2cj} = \sqrt{\frac{\sigma_2^2/(J_1 + J_c)}{2 \times MAF_{cj}(1 - MAF_{cj})}}\,.$$

U_1 and U_2 represent the set of confounding variables of the $X_1 - Y$ and $X_2 - Y$ associations. To ensure the confounders explained 25% of the variation in the risk factors, U_1 and U_2 were drawn independently from a normal distribution $\mathcal{N}(0, 0.25)$. To fix the variances of X_1 and X_2 to one, the error terms ϵ_1 and ϵ_2 were generated independently from a normal distribution

with mean zero, and variance:

$$\sigma^2_{\epsilon_1} = 1 - \sigma^2_1 - 0.25 \qquad \text{and} \qquad \sigma^2_{\epsilon_2} = 1 - \sigma^2_2 - 0.25\,.$$

The outcome Y was generated from:

$$Y_i = \theta_0 + \theta_1 X_{1i} + \theta_2 X_{2i} + \theta_{12} X_{12i} + 0.5U_{1i} + 0.5U_{2i} + \epsilon_{Yi}\,,$$

where θ_1 and θ_2 represent the main effects of X_1 and X_2 on Y, and θ_{12} represents the interaction effect of X_1 and X_2 on Y. X_{12} was generated by either: a) multiplying X_1 and X_2; or b) multiplying the mean centred values of the risk factors $(X_1 - \bar{X}_1)$ and $(X_2 - \bar{X}_2)$, where $\bar{X}_1$ and $\bar{X}_2$ are the mean values of X_1 and X_2. To ensure the risk factors and confounders explained less than a third of the variance in the outcome, the error term ϵ_Y was generated from a standard normal distribution $\mathcal{N}(0, 1)$.

Two-stage least squares regression models were fitted to either: a) the directly generated values of the risk factors (X_1, X_2, $X_{12} = X_1 \times X_2$); or b) the mean centred values of the risk factors ($X_1 - \bar{X}_1$, $X_2 - \bar{X}_2$, $X_{12} = (X_1 - \bar{X}_1) \times (X_2 - \bar{X}_2)$). When the risk factors were mean centred, the model estimated the marginal effects θ_{1M} and θ_{2M} of X_1 and X_2 on Y, otherwise θ_1 and θ_2 were estimated. For example, when there were no shared variants $J_c = 0$, the marginal effects were approximately:

$$\begin{aligned}\theta_{M1} &= \theta_1 + 0.3\theta_{12} + J_2\theta_{12}\left(\sqrt{\frac{0.1/J_2}{2 \times 0.3 \times 0.7}} \times 0.3 \times 2\right),\\ \theta_{M2} &= \theta_2 + 0.25\theta_{12} + J_1\theta_{12}\left(\sqrt{\frac{0.1/J_1}{2 \times 0.3 \times 0.7}} \times 0.3 \times 2\right).\end{aligned} \tag{A1}$$

The genetic variants were either treated as individual IVs or as a single instrument in externally weighted gene scores GS_{X_1} and GS_{X_2} for X_1 and X_2. The external weights for the gene scores were based on an independent set of 10 000 individuals, and were produced from the same data generating model used for the main set of participants. The following four sets of genetic variants were used as IVs in separate two-stage least squares regression models:

- Method 1 – full set of interactions: the J_1, J_2 and J_c genetic variants used to generate X_1 and X_2, plus the unique interactions and quadratic terms of $(\boldsymbol{G_1} + \boldsymbol{G_c}) \times (\boldsymbol{G_2} + \boldsymbol{G_c})$.
- Method 2 – reduced set of interactions: the J_1, J_2 and J_c genetic variants used to generate X_1 and X_2, plus the interactions from the product $\boldsymbol{G_1} \times \boldsymbol{G_2}$.
- Method 3 – continuous gene scores: the two weighted gene scores GS_{X_1} and GS_{X_2}, and their product $GS_{X_1} \times GS_{X_2}$.
- Method 4 – dichotomized gene scores: the two dichotomized gene scores, and their product.

Method 1 represents the oracle model as it includes all of the variables used in the data generating model, whereas Methods 2 to 4 are misspecified and their performance should be compared to Method 1. In Method 2, we have included a subset of the cross-terms between the genetic variants to create a more realistic scenario where the full set of relevant IVs are not included in the analysis. Method 3 considers the impact of including all of the genetic variants into two separate weighted gene scores, and finally, Method 4 considers the impact of dichotomizing the weighted gene scores.

Data were generated 10 000 times with $\theta_0 = 0.2$, $\theta_1 = 0.3$, $\theta_2 = 0.2$, and $\theta_{12} = 0.1, 0.3$ and 0.5. Each risk factor was associated with $(J_1 + J_c) = (J_2 + J_c) = 10$ genetic variants, and the number of shared variants J_c was initially set to 0 to consider the scenario where none of the genetic variants were associated with risk factors (Table 1). The data were re-generated for $\sigma_1^2 = \sigma_2^2 = 5\%$ and 1%, for $J_c = 0$ (Supplementary Table A1) and $J_c = 5$ (Supplementary Table A2), and the analyses were re-performed on the directly generated values of the risk factors. Estimates of the F-statistic and conditional F-statistic for X_1, X_2 and X_{12} were recorded. The analyses were re-performed on the mean centred risk factors (Supplementary Table A3), and the number of shared variants was set to $J_c = 1, 3, 5, 8$ and 10 (Table 2). The following measurements were recorded for the estimates of θ_1, θ_2 and θ_{12}: median estimate; standard deviation of estimates; median standard error of estimates; empirical power at the 5% significance level; and empirical coverage of the 95% confidence interval. The conditional F-statistic (also known as the Sanderson–Windmeijer F-statistic [1]) represents the strength of the IVs for the risk factors in a joint model, and is the relevant measure of instrument strength for a multivariable Mendelian randomization analysis [2].

	F-stat	CF-stat	Median	SD	Median SE	Power	Coverage
Variants explain 10% of the variance in risk factors:							
Methods 1 & 2 [a] – full set of interactions							
$\theta_1 = 0.3$	10.3 (0.6)	2.1 (0.3)	0.3043	0.0918	0.0910	91.0	95.0
$\theta_2 = 0.2$	10.3 (0.6)	2.1 (0.3)	0.2034	0.0947	0.0945	57.9	95.5
$\theta_{12} = 0.3$	8.1 (0.6)	1.9 (0.2)	0.3080	0.0722	0.0718	98.8	95.2
Method 3 – continuous gene scores							
$\theta_1 = 0.3$	364.2 (23.4)	104.5 (25.6)	0.2998	0.1359	0.1332	61.9	95.6
$\theta_2 = 0.2$	364.5 (23.2)	103.9 (25.3)	0.2019	0.1405	0.1387	31.5	95.8
$\theta_{12} = 0.3$	273.7 (22.4)	97.8 (22.8)	0.3000	0.1106	0.1091	77.5	95.8
Method 4 - dichotomized gene scores							
$\theta_1 = 0.3$	224.2 (17.7)	41.9 (13.4)	0.3039	0.2145	0.2074	32.1	95.8
$\theta_2 = 0.2$	224.4 (17.7)	41.7 (13.3)	0.2047	0.2236	0.2164	15.2	96.2
$\theta_{12} = 0.3$	168.2 (16.3)	40.0 (12.4)	0.2972	0.1777	0.1722	41.8	96.0
Variants explain 5% of the variance in risk factors:							
Methods 1 & 2 [a] – full set of interactions							
$\theta_1 = 0.3$	5.4 (0.4)	1.5 (0.2)	0.3174	0.0931	0.0920	92.4	94.5
$\theta_2 = 0.2$	5.4 (0.4)	1.4 (0.2)	0.2166	0.0957	0.0959	62.0	94.8
$\theta_{12} = 0.3$	3.9 (0.4)	1.2 (0.2)	0.3087	0.0889	0.0888	92.8	95.0
Method 3 – continuous gene scores							
$\theta_1 = 0.3$	170.2 (15.5)	25.4 (11.7)	0.2988	0.2298	0.2121	29.9	96.9
$\theta_2 = 0.2$	170.1 (15.7)	25.2 (11.5)	0.1985	0.2421	0.2237	13.8	96.9
$\theta_{12} = 0.3$	109.4 (13.3)	23.8 (10.4)	0.3020	0.2458	0.2276	26.7	96.9
Method 4 - dichotomized gene scores							
$\theta_1 = 0.3$	107.3 (12.2)	10.7 (6.7)	0.2970	3.928	0.3367	12.6	98.9
$\theta_2 = 0.2$	106.9 (12.0)	10.6 (6.6)	0.1948	3.804	0.3551	5.4	98.7
$\theta_{12} = 0.3$	68.8 (10.2)	10.2 (6.1)	0.3033	4.065	0.3654	10.8	98.8
Variants explain 1% of the variance in risk factors:							
Methods 1 & 2 [a] – full set of interactions							
$\theta_1 = 0.3$	1.8 (0.2)	1.4 (0.2)	0.3681	0.0910	0.0901	97.7	88.4
$\theta_2 = 0.2$	1.8 (0.2)	1.4 (0.2)	0.2670	0.0930	0.0930	81.4	88.6
$\theta_{12} = 0.3$	1.4 (0.2)	1.0 (0.1)	0.3029	0.0971	0.0972	86.4	95.4
Method 3 – continuous gene scores							
$\theta_1 = 0.3$	29.5 (6.4)	1.9 (2.9)	0.2854	29.26	0.8411	2.8	99.9
$\theta_2 = 0.2$	29.4 (6.4)	1.9 (2.8)	0.1883	31.58	0.9203	1.0	99.9
$\theta_{12} = 0.3$	12.3 (4.1)	1.6 (2.1)	0.3185	52.32	1.537	0.7	100.0
Method 4 - dichotomized gene scores							
$\theta_1 = 0.3$	19.1 (5.1)	1.6 (2.8)	0.2992	123.8	1.063	1.9	99.9
$\theta_2 = 0.2$	19.0 (5.0)	1.5 (2.4)	0.1930	217.5	1.163	0.6	100.0
$\theta_{12} = 0.3$	8.1 (3.3)	1.2 (1.7)	0.3121	347.4	1.933	0.3	100.0

Supplementary Table A1: Simulation study results for interactions between risk factors varying the amount of variance in the risk factors explained by the genetic variants, with no shared variants and an interaction effect $\theta_{12} = 0.3$: mean F-statistic (F-stat), mean conditional F-statistic (CF-stat), median estimate, standard deviation (SD) of estimates, median standard error (SE), empirical power (%) to reject null at 5% significance, and empirical coverage (%) of 95% confidence interval.

[a]As there are no shared variants, methods 1 and 2 are equivalent.

	F-stat	CF-stat	Median	SD	Median SE	Power	Coverage
Variants explain 10% of the variance in risk factors:							
Method 1 – full set of interactions							
$\theta_1 = 0.3$	11.6 (0.7)	2.5 (0.4)	0.2981	0.0933	0.0927	89.1	95.0
$\theta_2 = 0.2$	11.6 (0.7)	2.5 (0.4)	0.1988	0.0955	0.0960	55.0	95.5
$\theta_{12} = 0.3$	13.4 (0.9)	2.2 (0.3)	0.3074	0.0707	0.0706	99.0	95.0
Method 2 – reduced set of interactions							
$\theta_1 = 0.3$	28.8 (1.8)	2.6 (0.4)	0.2970	0.1664	0.1649	44.2	95.8
$\theta_2 = 0.2$	28.8 (1.8)	2.6 (0.4)	0.1966	0.1719	0.1715	21.0	95.9
$\theta_{12} = 0.3$	32.6 (2.1)	2.3 (0.3)	0.3056	0.1337	0.1333	63.4	95.8
Method 3 – continuous gene scores							
$\theta_1 = 0.3$	366.4 (23.2)	131.8 (30.9)	0.2993	0.1272	0.1244	67.0	95.4
$\theta_2 = 0.2$	366.3 (23.4)	131.0 (30.7)	0.1992	0.1314	0.1293	35.1	95.4
$\theta_{12} = 0.3$	426.6 (29.1)	120.9 (26.9)	0.3008	0.1000	0.0978	84.8	95.4
Method 4 - dichotomized gene scores							
$\theta_1 = 0.3$	233.5 (18.1)	35.8 (12.4)	0.2984	0.2399	0.2302	25.9	96.4
$\theta_2 = 0.2$	233.5 (18.2)	35.6 (12.3)	0.2005	0.2482	0.2396	13.0	96.4
$\theta_{12} = 0.3$	284.1 (21.6)	33.8 (11.2)	0.3006	0.1950	0.1877	36.8	96.4
Variants explain 5% of the variance in risk factors:							
Method 1 – full set of interactions							
$\theta_1 = 0.3$	6.0 (0.5)	1.6 (0.2)	0.3052	0.0980	0.0983	87.7	95.2
$\theta_2 = 0.2$	6.0 (0.5)	1.5 (0.2)	0.2078	0.1018	0.1022	53.3	95.2
$\theta_{12} = 0.3$	6.1 (0.5)	1.3 (0.2)	0.3097	0.0925	0.0919	91.3	95.3
Method 2 – reduced set of interactions							
$\theta_1 = 0.3$	14.2 (1.2)	1.6 (0.3)	0.2982	0.1600	0.1588	48.4	96.3
$\theta_2 = 0.2$	14.2 (1.2)	1.6 (0.3)	0.1994	0.1665	0.1664	22.7	96.1
$\theta_{12} = 0.3$	13.9 (1.3)	1.4 (0.2)	0.3087	0.1621	0.1615	49.0	96.1
Method 3 – continuous gene scores							
$\theta_1 = 0.3$	171.8 (15.6)	32.9 (14.1)	0.3014	0.2078	0.1951	35.7	96.4
$\theta_2 = 0.2$	172.1 (15.4)	32.6 (13.9)	0.2041	0.2169	0.2043	16.9	96.5
$\theta_{12} = 0.3$	171.7 (17.6)	30.0 (12.0)	0.2981	0.2147	0.2010	32.6	96.5
Method 4 - dichotomized gene scores							
$\theta_1 = 0.3$	111.9 (12.5)	9.5 (6.4)	0.2933	0.8024	0.3732	10.2	99.1
$\theta_2 = 0.2$	112.2 (12.3)	9.4 (6.3)	0.1981	0.8127	0.3926	4.6	98.9
$\theta_{12} = 0.3$	117.6 (13.5)	8.9 (5.7)	0.3066	0.8619	0.3967	9.6	99.1
Variants explain 1% of the variance in risk factors:							
Method 1 – full set of interactions							
$\theta_1 = 0.3$	2.0 (0.2)	1.4 (0.2)	0.3504	0.0975	0.0971	94.4	92.0
$\theta_2 = 0.2$	2.0 (0.2)	1.3 (0.2)	0.2478	0.1003	0.1002	69.6	92.2
$\theta_{12} = 0.3$	1.6 (0.2)	1.0 (0.1)	0.3037	0.1051	0.1043	82.0	95.3
Method 2 – reduced set of interactions							
$\theta_1 = 0.3$	3.5 (0.6)	1.4 (0.2)	0.3225	0.1398	0.1395	63.8	95.6
$\theta_2 = 0.2$	3.5 (0.5)	1.4 (0.2)	0.2243	0.1459	0.1457	34.3	95.7
$\theta_{12} = 0.3$	2.6 (0.5)	1.1 (0.1)	0.3036	0.1771	0.1758	41.8	96.1
Method 3 – continuous gene scores							
$\theta_1 = 0.3$	31.0 (6.6)	2.5 (3.7)	0.2912	47.33	0.7448	3.6	99.9
$\theta_2 = 0.2$	30.9 (6.5)	2.3 (3.4)	0.1939	41.15	0.8014	1.1	99.9
$\theta_{12} = 0.3$	19.9 (5.4)	1.9 (2.4)	0.3030	72.69	1.315	0.6	99.9
Method 4 - dichotomized gene scores							
$\theta_1 = 0.3$	20.9 (5.3)	1.6 (2.9)	0.2967	65.97	1.108	1.5	99.9
$\theta_2 = 0.2$	20.8 (5.2)	1.5 (2.5)	0.1959	54.84	1.208	0.4	100.0
$\theta_{12} = 0.3$	14.1 (4.4)	1.2 (1.6)	0.3096	105.7	1.991	0.2	100.0

Supplementary Table A2: Simulation study results for interactions between risk factors varying the amount of variance in the risk factors explained by the genetic variants, with 5 shared variants and an interaction effect $\theta_{12} = 0.3$: mean F-statistic (F-stat), mean conditional F-statistic (CF-stat), median estimate, standard deviation (SD) of estimates, median standard error (SE), empirical power (%) to reject null at 5% significance, and coverage (%) of 95% confidence interval.

	Median	SD	Median SE	Power (%)	Coverage (%)
Methods 1 & 2 [a] – full set of interactions					
$\theta_1 = 0.3$	0.4311	0.0327	0.0320	100.0	-
$\theta_2 = 0.2$	0.3370	0.0328	0.0320	100.0	-
$\theta_{12} = 0.1$	0.1101	0.0721	0.0718	33.7	94.6
$\theta_1 = 0.3$	0.6679	0.0408	0.0320	100.0	-
$\theta_2 = 0.2$	0.5823	0.0413	0.0320	100.0	-
$\theta_{12} = 0.3$	0.3080	0.0722	0.0718	98.8	95.2
$\theta_1 = 0.3$	0.9044	0.0527	0.0320	100.0	-
$\theta_2 = 0.2$	0.8290	0.0528	0.0320	100.0	-
$\theta_{12} = 0.5$	0.5073	0.0715	0.0718	100.0	95.2
Method 3 – continuous gene scores					
$\theta_1 = 0.3$	0.4178	0.0348	0.0343	100.0	-
$\theta_2 = 0.2$	0.3234	0.0349	0.0343	100.0	-
$\theta_{12} = 0.1$	0.1010	0.1113	0.1091	15.4	95.5
$\theta_1 = 0.3$	0.6539	0.0424	0.0343	100.0	-
$\theta_2 = 0.2$	0.5691	0.0431	0.0343	100.0	-
$\theta_{12} = 0.3$	0.3000	0.1106	0.1091	77.5	95.8
$\theta_1 = 0.3$	0.8906	0.0539	0.0343	100.0	-
$\theta_2 = 0.2$	0.8165	0.0543	0.0343	100.0	-
$\theta_{12} = 0.5$	0.4995	0.1107	0.1092	98.7	95.6
Method 4 – dichotomized gene scores					
$\theta_1 = 0.3$	0.4173	0.0438	0.0435	100.0	-
$\theta_2 = 0.2$	0.3236	0.0438	0.0434	100.0	-
$\theta_{12} = 0.1$	0.1022	0.1786	0.1720	8.0	95.9
$\theta_1 = 0.3$	0.6538	0.0496	0.0435	100.0	-
$\theta_2 = 0.2$	0.5687	0.0506	0.0435	100.0	-
$\theta_{12} = 0.3$	0.2972	0.1777	0.1722	41.8	96.0
$\theta_1 = 0.3$	0.8913	0.0597	0.0435	100.0	-
$\theta_2 = 0.2$	0.8165	0.0603	0.0435	100.0	-
$\theta_{12} = 0.5$	0.5002	0.1776	0.1718	80.7	96.1

Supplementary Table A3: Simulation study results for interactions between risk factors with no shared variants after centering the risk factors: median estimate, standard deviation (SD) of estimates, median standard error (SE), empirical power (%) to reject null at 5% significance, and empirical coverage (%) of 95% confidence interval. Note that centering changes the estimands for the main effect terms, not only the estimates – hence coverage is only displayed for the interaction term.

[a] As there are no shared variants, methods 1 and 2 are equivalent.

Simulation study 2: interactions between interventions

Using the same notation defined in the first simulation study, the risk factor X was generated for $i = 1, 2, \ldots, 10\,000$ participants from the following data generating model:

$$X_i = 0.3 + \sum_{j=1}^{J_A} \alpha_{Aj} G_{Aji} + \sum_{j=1}^{J_B} \alpha_{Bj} G_{Bji} + \alpha_{AB} \sum_{j=1}^{J_A \times J_B} G_{ABji} + U_i + \epsilon_{Xi}\,.$$

We assume that the two gene regions are distinct, and the genetic variants $\boldsymbol{G_A}$ and $\boldsymbol{G_B}$ are not in linkage disequilibrium. The genotypes were generated independently from binomial distributions $\text{Bin}(2, MAF_j)$, where MAF_j represents the MAF for the jth genetic variant. MAF_j was drawn from a uniform distribution $\mathcal{U}(MAF_L, MAF_U)$, where the value of MAF_L and MAF_U were either taken as 0.4 and 0.5 (common variants), or 0.1 and 0.2 (uncommon variants). We assumed that the interaction effect α_{AB} was constant across the $J_A \times J_B$ product terms for simplicity.

The approximate proportion of variance explained in X by $\boldsymbol{G_A}$ (σ_A^2) and $\boldsymbol{G_B}$ (σ_B^2) varied between scenarios. As before, the genetic associations $\boldsymbol{\alpha_A}$ and $\boldsymbol{\alpha_B}$ were calculated by rearranging the formula for the variance of the genetic variants to ensure the amount of variance explained by each variant was the same:

$$\alpha_{Aj} = \sqrt{\frac{\sigma_A^2/J_A}{2 \times MAF_{Aj}(1 - MAF_{Aj})}} \quad \text{and}$$
$$\alpha_{Bj} = \sqrt{\frac{\sigma_B^2/J_B}{2 \times MAF_{Bj}(1 - MAF_{Bj})}}\,.$$

The confounders U were drawn from $\mathcal{N}(0, 0.25)$, and the error term ϵ_X was generated from $\mathcal{N}(0, 0.65)$. The outcome Y was generated from:

$$Y_i = \theta_0 + \theta_1 X_i + U_i + \epsilon_{Yi}\,,$$

where θ_1 represents the causal effect of X on Y, and the error term ϵ_Y was generated from a standard normal distribution $\mathcal{N}(0, 1)$. The data was generated 10 000 times under the following scenarios:

- Scenario 1: $MAF_A \sim \mathcal{U}(0.4, 0.5)$, $MAF_B \sim \mathcal{U}(0.4, 0.5)$, $\sigma_A^2 = 3\%$ and $\sigma_B^2 = 3\%$
- Scenario 2: $MAF_A \sim \mathcal{U}(0.4, 0.5)$, $MAF_B \sim \mathcal{U}(0.4, 0.5)$, $\sigma_A^2 = 5\%$ and $\sigma_B^2 = 5\%$
- Scenario 3: $MAF_A \sim \mathcal{U}(0.4, 0.5)$, $MAF_B \sim \mathcal{U}(0.4, 0.5)$, $\sigma_A^2 = 3\%$ and $\sigma_B^2 = 7\%$
- Scenario 4: $MAF_A \sim \mathcal{U}(0.1, 0.2)$, $MAF_B \sim \mathcal{U}(0.1, 0.2)$, $\sigma_A^2 = 5\%$ and $\sigma_B^2 = 5\%$
- Scenario 5: $MAF_A \sim \mathcal{U}(0.4, 0.5)$, $MAF_B \sim \mathcal{U}(0.1, 0.2)$, $\sigma_A^2 = 5\%$ and $\sigma_B^2 = 5\%$
- Scenario 6: $MAF_A \sim \mathcal{U}(0.4, 0.5)$, $MAF_B \sim \mathcal{U}(0.1, 0.2)$, $\sigma_A^2 = 3\%$ and $\sigma_B^2 = 7\%$

with $J_A = J_B = 3$, $\theta_0 = 0.2$, $\theta_1 = 0.1$, and $\alpha_{AB} = 0.1, 0.3$ and 0.5. The above scenarios were selected to consider the impact of varying the MAF and the amount of variance in the risk factor explained by the genetic variants had on the performance of the method.

For each scenario, optimal weighted gene scores GS_A and GS_B were generated for each gene region, where the external weights were produced from an independent set of 10 000

individuals from the same data-generating model used for the main set of participants. The two gene scores were dichotomized at their median values to create two binary variables. The outcome was then regressed against: a) the two continuous gene scores and their product; and b) the dichotomized gene scores and their product. The following measurements were recorded for the estimate of the interaction effect between the gene scores on the outcome: median estimate; standard deviation of estimates; median standard error; and empirical power at the 5% significance level.

Applied example: the effects of BMI and alcohol on systolic blood pressure

UK Biobank is a prospective, population-based cohort consisting of approximately 500,000 participants aged between 40 and 69 years at baseline living in the UK. Extensive baseline characteristics were collected at recruitment, including lifestyle factors, sociodemographic information, and physical attributes. For the analysis, we considered 367,643 unrelated participants of European descent who passed data quality control measures and had genetic data [3].

Body mass index (BMI, kg/m^2) and systolic blood pressure (SBP, mmHg) were measured at baseline when participants attended the assessment centre. Information on baseline alcohol consumption was obtained from a touchscreen questionnaire which included questions on alcohol drinking status, frequency of alcohol consumption, and beverage type. The responses to the amount of alcohol drank and beverage type were used to create a continuous variable that represented alcohol consumption in units per day. To adjust for blood pressure medication, 15 mmHg was added to SBP for individuals who reported to be on blood pressure lowering medication [4]. Individuals were dropped from the analysis if they had missing data on BMI, SBP, alcohol consumption, or relevant genetic variants. The final sample size was 291,781.

We used the 77 genome-wide significant variants from a meta-analysis by the Genetic Investigation of ANthropometric Traits (GIANT) consortium in participants of European ancestry to act as IVs for BMI [5]. For alcohol, we identified 10 genetic variants in the *ADH1B* gene region that have been shown to be associated with alcohol consumption [6]. The genetic variants used as IVs for BMI and alcohol consumption were cross-referenced to check for any overlap. BMI was regressed separately against each of the 10 alcohol variants, and alcohol consumption was regressed against each of the 77 BMI variants. All models were adjusted for gender, age, and the first ten genomic principal components.

Internally-weighted gene scores were created for BMI based on the 77 genetic variants (GS_{BMI}), and for alcohol consumption based on the 10 genetic variants (GS_{AC}), and these gene scores were dichotomized at their median values to create two binary variables. A separate binary variable was generated using the rs1229984 variant only, where participants were either considered to have: a) a low alcohol consumption if they were homozygous or heterozygous for the alcohol-decreasing allele; or b) a high alcohol consumption if they were homozygous for the alcohol-increasing allele (as in the paper by Carter *et al.* [7]). Using these binary variables, the following groups of participants were created:

- Low BMI, low alcohol consumption: $GS_{BMI} \leq med(GS_{BMI})$ and $GS_{AC} \leq med(GS_{AC})$ or was homozygous or heterozygous for the alcohol decreasing allele for the rs1229984 variant,
- High BMI, low alcohol consumption: $GS_{BMI} > med(GS_{BMI})$ and $GS_{AC} \leq med(GS_{AC})$ or was homozygous or heterozygous for the alcohol decreasing allele for the rs1229984 variant,
- Low BMI, high alcohol consumption: $GS_{BMI} \leq med(GS_{BMI})$ and $GS_{AC} > med(GS_{AC})$ or was homozygous for the alcohol increasing allele for the rs1229984 variant, and
- High BMI, high alcohol consumption: $GS_{BMI} \leq med(GS_{BMI})$ and $GS_{AC} > med(GS_{AC})$ or was homozygous for the alcohol increasing allele for the rs1229984 variant.

The above criteria created four groups of participants based on the dichotomized gene scores for BMI and alcohol consumption, and another four groups based on the dichotomized gene

score for BMI and the rs1229984 variant. The numbers of participants, and the mean and standard deviation of BMI, alcohol consumption, and SBP were recorded for each group.

Two-stage least squares regression models of SBP were fitted to BMI, alcohol consumption, and the product of BMI and alcohol consumption. The following sets of IVs were considered:

- Method 1: the 77 variants for BMI and 10 variants for alcohol consumption, plus 770 cross-terms between the two sets of variants.
- Method 2: the continuous gene scores GS_{BMI} and GS_{AC}, plus their product $GS_{BMI} \times GS_{AC}$.
- Method 3: the dichotomized gene scores of GS_{BMI} and GS_{AC}, plus their product.

The models were refitted excluding all of the variants for alcohol consumption apart from the lead rs1229984 variant. All models were adjusted for gender, age, and the first ten genomic principal components. For each model, the estimate and standard error of the interaction term was recorded with its p-value. In total, six two-stage least squares regression models were fitted to the dataset, and all of the models were adjusted for age, gender and the first 10 genomic principal components. The F-statistic and the Sanderson–Windmeijer conditional F-statistic were estimated for each set of IVs with respect to BMI, alcohol consumption, and the product of BMI and alcohol consumption (Supplementary Table A4).

	Method 1		Method 2		Method 3	
	F-stat	CF-stat	F-stat	CF-stat	F-stat	CF-stat
10 variants for alcohol:						
BMI	6.8	1.3	1662.8	21.1	1054.1	7.0
Alcohol consumption	2.4	1.1	268.0	20.9	55.6	6.9
Product term	2.4	1.1	298.6	21.0	73.2	6.9
rs1229984 for alcohol:						
BMI	32.8	1.3	1654.9	17.2	1066.8	13.5
Alcohol consumption	7.7	1.2	245.1	17.1	241.6	13.4
Product term	7.9	1.2	267.7	17.1	266.5	13.4

Supplementary Table A4: F-statistics (F-stat) and conditional F-statistics (CF-stat) for applied example.

Supplementary References

[1] Sanderson E, Windmeijer F. A weak instrument F-test in linear IV models with multiple endogenous variables. Journal of Econometrics. 2016;190(2):212–221.

[2] Sanderson E, Davey Smith G, Windmeijer F, Bowden J. An examination of multivariable Mendelian randomization in the single sample and two-sample summary data settings. International Journal of Epidemiology. 2018;.

[3] Astle WJ, Elding H, Jiang T, Allen D, Ruklisa D, Mann AL, et al. The allelic landscape of human blood cell trait variation and links to common complex disease. Cell. 2016;167(5):1415–1429.

[4] Warren HR, Evangelou E, Cabrera CP, Gao H, Ren M, Mifsud B, et al. Genome-wide association analysis identifies novel blood pressure loci and offers biological insights into cardiovascular risk. Nature Genetics. 2017;49(3):403–415.

[5] Locke AE, Kahali B, Berndt SI, Justice AE, Pers TH, Day FR, et al. Genetic studies of body mass index yield new insights for obesity biology. Nature. 2015;518(7538):197–206.

[6] Lewis SJ, Zuccolo L, Smith GD, Macleod J, Rodriguez S, Draper ES, et al. Fetal alcohol exposure and IQ at age 8: evidence from a population-based birth-cohort study. PLOS One. 2012;7(11):e49407.

[7] Carter AR, Borges MC, Benn M, Tybjærg-Hansen A, Davey Smith G, Nordestgaard BG, et al. Combined association of body mass index and alcohol consumption with biomarkers for liver injury and incidence of liver disease: a Mendelian randomization study. JAMA Network Open. 2019;2(3):e190305.
